# Carbohydrate Quality Versus Quantity in Gestational Diabetes Mellitus While Considering Randomized Dietary Interventions: A Systematic Review

**DOI:** 10.7759/cureus.117076

**Published:** 2026-09-28

**Authors:** Swati Aggarwal, Gurjeet K Chawla, Renu Gupta

**Affiliations:** 1 Nutrition and Dietetics, Manav Rachna International Institute of Research and Studies, Faridabad, IND; 2 Gynecology and Obstetrics, Sri Balaji Action Medical Institute, New Delhi, IND

**Keywords:** carbohydrate, dietary fiber, gestational diabetes mellitus, glycemic index, systematic review

## Abstract

Medical nutrition therapy is the first-line treatment for gestational diabetes mellitus (GDM), but whether carbohydrate quality or quantity should be prioritized is unclear, as dietary interventions often change amount, glycemic quality, fiber, fat, protein, and food pattern together. We systematically reviewed randomized trials of carbohydrate quality or quantity in diagnosed GDM. PubMed and the Cochrane Central Register of Controlled Trials (CENTRAL) were searched from inception to July 19, 2026, with citation searching. Two human reviewers screened the records and extracted the data with artificial-intelligence assistance. Risk of bias was assessed using the revised Cochrane risk-of-bias tool (RoB 2), and the findings were primarily synthesized narratively using the Synthesis Without Meta-analysis (SWiM) approach, with limited random-effects meta-analysis for clinically compatible insulin-related outcomes. From 981 records, 34 reports representing 25 independent randomized studies were included; they differed markedly in construct, achieved carbohydrate separation, rescue treatment, and analysis population. In an exploratory, treatment-threshold analysis, lower-glycemic-index assignment reduced the proportion meeting insulin-start criteria (two trials, 159 participants; risk ratio [RR] 0.43, 95% confidence interval [CI] 0.25-0.75), but rescue switching removed the difference in actual insulin use. Lower-carbohydrate assignment did not reduce insulin initiation (three trials, 248 participants; RR 1.02, 95% CI 0.76-1.35). This finding remained essentially unchanged after excluding the trial in which the intended difference in carbohydrate intake was not achieved. With only two or three small trials per pool, I² = 0% does not indicate homogeneity. Fiber, staple-quality, and provided-diet interventions improved selected postprandial measures, but obstetric, birth-size, and neonatal-adiposity benefits were not established, and certainty was low or very low. Carbohydrate quality is a plausible route to postprandial improvement without aggressive restriction, but current randomized evidence does not establish a universal quality-over-quantity rule or support lower carbohydrate intake as superior. Carbohydrate amount should be individualized while prioritizing nutrient-dense, lower-glycemic-index, fiber-rich sources.

## Introduction and background

Medical nutrition therapy (MNT) is the first-line treatment for most women with gestational diabetes mellitus (GDM), but the optimal carbohydrate prescription remains unsettled. Guidance emphasizes adequate nutrition, avoidance of severe restriction, and individualized glycemic control; the 2026 American Diabetes Association Standards of Care reiterate a minimum of 175 g of carbohydrate daily in pregnancy but do not specify which carbohydrate foods to eat or whether lowering the energy percentage from carbohydrate improves maternal or neonatal outcomes [1,2]. Previous evidence suggests that dietary modification in GDM may improve glycemic control, but findings for maternal and neonatal outcomes, such as gestational weight gain, hypertensive disorders, birth weight, large-for-gestational-age birth, and neonatal adiposity, have been inconsistent. Differences in carbohydrate quality, quantity, dietary composition, intervention duration, and outcome definitions make it difficult to determine whether one carbohydrate strategy provides consistent pregnancy or neonatal benefits [3].

Carbohydrate quantity (grams or percentage of energy) and quality (glycemic index [GI], glycemic load [GL], fiber, food structure, processing, and source) are distinct exposures. A diet based on lower-GI carbohydrate sources can maintain a similar amount of carbohydrate, whereas reducing carbohydrate intake generally increases the relative contribution of fat or protein; many trials change both, and some also alter energy or counseling intensity; thus, effects cannot be attributed to carbohydrate alone unless prescribed, and achieved intakes are reported [3].

A 2024 meta-analysis by Wong et al. pooled broader dietary groupings [3]. We instead worked from study-level records, excluded evidence syntheses as primary studies, mapped companion reports to independent trial families, and assessed risk of bias at the outcome level to address a narrower question: what do randomized comparisons show about carbohydrate quality and quantity after GDM diagnosis, and which effects are comparable enough to pool? We hypothesized that quality-focused strategies could improve postprandial control without a consistent advantage from greater restriction, while recognizing that heterogeneity might preclude broad meta-analysis.

## Review

Methods

Design, Reporting, and Eligibility

This review followed PRISMA (Preferred Reporting Items for Systematic Reviews and Meta-Analyses) 2020 and Cochrane methods, with narrative synthesis per the Synthesis Without Meta-analysis (SWiM) guideline, and was not prospectively registered [4-6]. Eligible participants were pregnant women with diagnosed GDM (mixed populations only when GDM results were separable), and eligible designs were randomized parallel or crossover dietary trials and their companion reports. Eligible interventions changed carbohydrate quality (GI, GL, fiber, whole-grain or staple quality, or processing), quantity (grams or energy percentage), or a defined mixed pattern; acute test meals were retained as mechanistic evidence but analyzed separately. Systematic reviews, observational studies, quasi-random allocation, meal-timing-only interventions, and trials without an eligible carbohydrate contrast were excluded. Outcomes spanned self-monitored and continuous glucose monitoring (SMBG/CGM), fasting and postprandial glucose, insulin sensitivity, medication, ketones, maternal weight and lipids, hypertensive and delivery outcomes, birth size, neonatal adiposity and morbidity, adherence, safety, and postpartum outcomes.

Information Sources and Search

PubMed/MEDLINE and the Cochrane Central Register of Controlled Trials (CENTRAL) were searched from inception to July 19, 2026, without language or date limits, combining GDM terms with carbohydrate, GI, fiber, whole-grain, starch, and diet or meal terms. Google Scholar was used only for supplementary discovery because its results were unstable and not reproducibly exportable. Backward and forward citation searching and reference checking of eligible reports and of Wong et al. added nine unique records; full line-by-line strategies and yields are provided in Appendix A [3].

Selection, Report Linkage, and Artificial Intelligence Assistance

The search was conducted from inception through July 19, 2026, to capture studies published or indexed up to the search date. No additional eligible randomized reports published or indexed in 2026 before the search cutoff were identified through the database and supplementary searches. Two human reviewers independently screened and assessed full texts, resolving disagreements by discussion. Artificial-intelligence tools assisted bibliographic organization, extraction from supplied full texts, consistency checking, and formatting but did not determine eligibility, data interpretation, trial-family assignment, risk-of-bias judgments, poolability, statistical decisions, or the conclusions, which were made and verified by the reviewers. Multiple reports were linked by registry, authorship, participants, dates, intervention, and sample size before studies or participants were counted.

Data Extraction and Reconciliation

Extraction captured design, GDM criteria, randomized and analyzed denominators, intervention and comparator, duration, prescribed and achieved carbohydrate, GI/GL/fiber, macronutrient and energy changes, food provision, adherence, rescue treatment, outcome definitions, analysis population, and source location. Source errors and arithmetic discrepancies (for example, mixed standard-deviation and standard-error reporting and discrepant CHOICE flow-diagram arithmetic) were preserved in a correction ledger.

Intervention Taxonomy, Risk of Bias, and Synthesis

Trials were grouped by intended construct and interpreted using achieved intake, with failure to achieve the prescribed separation treated as implementation failure rather than equivalence. RoB 2 was applied to specific outcomes across its five domains, including the crossover extension where relevant [7].

Pooling required a comparable construct, outcome definition, timepoint, and analysis population. Binary outcomes are reported as risk ratios (RRs) with 95% confidence intervals (CIs) from DerSimonian-Laird random-effects models, with a 0.5 continuity correction for a single zero-event arm; a sensitivity analysis excluded the lower-carbohydrate trial that achieved no meaningful carbohydrate difference. With only two or three studies per pool, I² was reported but not interpreted as reliable, and a value of 0% was not taken as evidence of homogeneity. Crossover outcomes were not pooled without paired variances. Grading of Recommendations Assessment, Development and Evaluation (GRADE) certainty was assessed for each major body of evidence (full profile in Appendix B), and publication-bias tests were not used because no pool reached 10 studies [8].

Study selection

The searches identified 972 database records (PubMed, 616; CENTRAL, 356) and nine records by other methods. After removal of 125 duplicates, 856 records were screened and 807 were excluded. Forty-nine reports were sought; five could not be retrieved. Forty-four full texts were assessed, 10 were excluded (wrong population, n = 3; wrong intervention, n = 3; wrong design, n = 1; ineligible comparison, n = 1; ongoing or no results, n = 2), and 34 reports representing 25 independent randomized studies were included (Figure 1).

**Figure 1:**
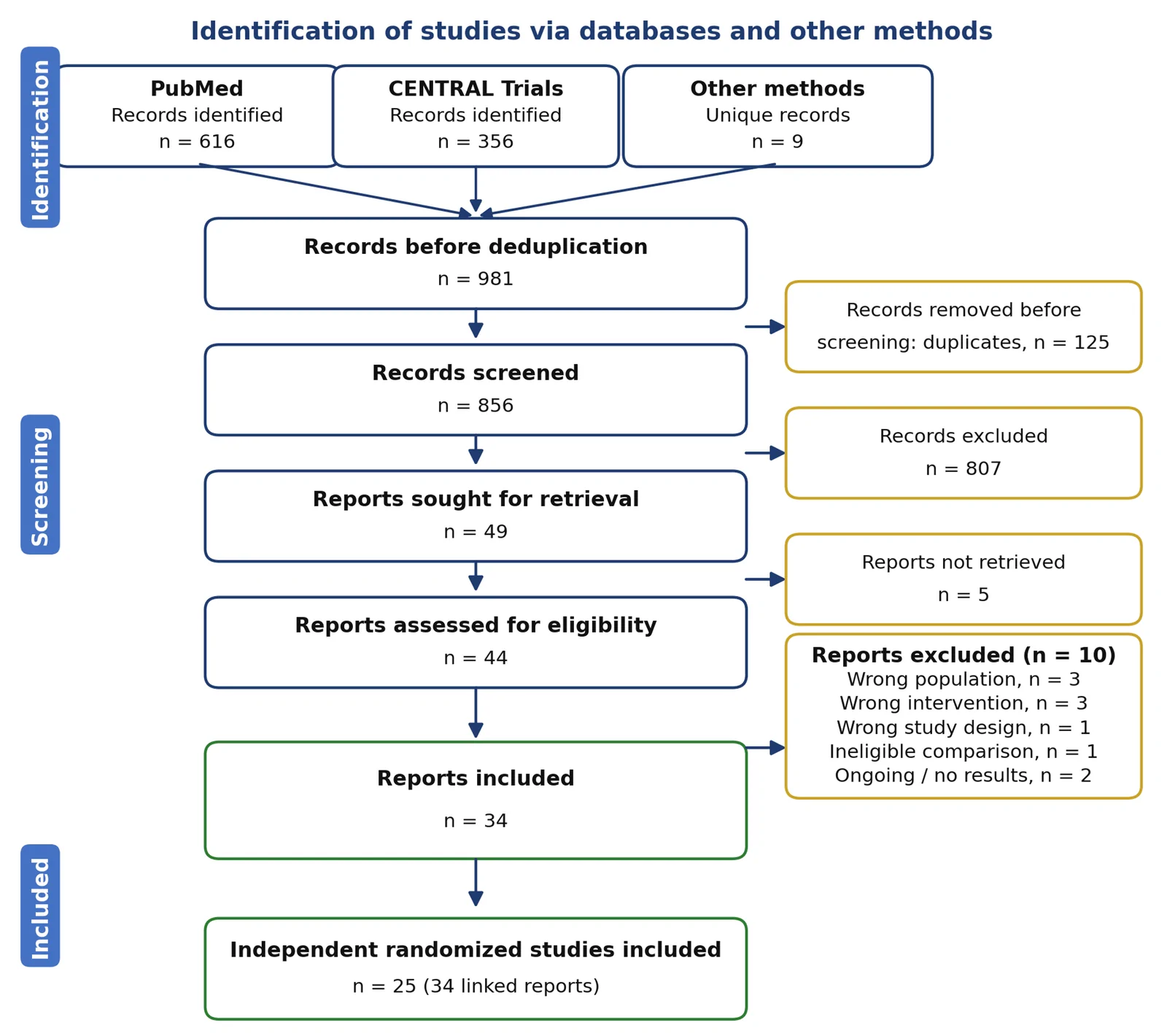
PRISMA 2020 flow diagram of study identification, screening, and inclusion Reports are the selection unit; trial families are the independent study unit. PRISMA, Preferred Reporting Items for Systematic Reviews and Meta-Analyses; CENTRAL, Cochrane Central Register of Controlled Trials

Included Reports, Trial Families, and Intervention Heterogeneity

Table 1 presents the 34 included reports and their linkage to 25 independent trial families (24 primary or phase reports, 5 companion reports, and 5 conference abstracts), published in 1984-2025 across Asia, Europe, Australia, and the United States [9-42]. Trial-family sizes ranged from 5 (Nolan) to 224 (Duraiswamy et al.); 19 families were parallel-group, 4 were acute or short-crossover feeding studies, and 2 combined a crossover phase with a longer parallel phase, with durations from a single meal or 36-hour feeding to continuation until delivery [9,14,18,24,30,33,35]. The included trials were organized into quality-focused, quantity-focused, and mixed dietary interventions. Quality-focused trials evaluated GI, GL, fiber, grain or staple quality, or food processing, whereas quantity-focused trials altered carbohydrate grams or percentage of energy, often with reciprocal changes in fat or protein. Mixed interventions changed carbohydrate together with other macronutrients or the overall dietary pattern and therefore could not be interpreted as effects of carbohydrate alone. Detailed intervention characteristics and study findings are provided in Table 1 and Appendix C.

**Table 1:** Characteristics of the 25 independent randomized trial families Construct denotes the primary carbohydrate exposure the trial was designed to test: quality (GI, GL, fiber, or staple composition), quantity (grams or percentage of energy), or a mixed/whole-diet change. “Participants (randomized; analyzed)” gives the number randomized followed by the number contributing to the reported analysis; counts are not summed across linked reports or phases. CHO, carbohydrate; CGM, continuous glucose monitoring; DASH, Dietary Approaches to Stop Hypertension; GI, glycemic index; GL, glycemic load; ITT, intention-to-treat; mITT, modified intention-to-treat; MNT, medical nutrition therapy; MUFA, monounsaturated fatty acid

Trial family (references)	Country	Design	Participants (randomized; analyzed)	Construct	Intervention	Comparator	Duration
Nolan, 1984 [9]	Australia	Crossover	5; 4	Mixed	70% CHO, high unrefined fiber, 10% fat	35% CHO, 45% fat	4 days per crossover period
Lauszus et al., 2001 [10]	Denmark	Parallel	27; 27	Quantity/substitution	High-MUFA diet	High-carbohydrate diet	5-7 weeks
Cypryk et al., 2007 [11]	Poland	Parallel	30; 30	Quantity	~45% energy from CHO	~65% energy from CHO	14 days, then to delivery
Moses et al., 2009 [12]	Australia	Parallel	63; 63	Quality	Low-GI diet	High-fiber, higher-GI diet	~7-10 wk
Louie et al., 2011/2015 [13,15]	Australia	Parallel + postpartum follow-up	99; 92 pregnancy, 58 postpartum	Quality	Low-GI diet	High-fiber, moderate-GI diet	6-7 wk; 3-mo follow-up
Louie et al., 2013 [14]	Australia	Crossover (single meals)	10; 8 paired	Quality	GI 45 breakfast (CHO-matched)	GI 82 breakfast (CHO-matched)	Single meals
Afaghi et al., 2013 [16]	Iran	Parallel	36; 31	Quality/fiber	Low-GL diet + wheat bran	Low-GL diet alone	2 wk
Moreno-Castilla et al., 2013 [17]	Spain	Parallel	152; 150 (mITT)	Quantity	40% energy from CHO	55% energy from CHO	~9 wk
Hernandez et al., 2014/2016 (CHOICE pilot) [18,21,38,39]	USA	Crossover, then continuation	19 crossover; 16/12 continuation	Mixed	60% complex CHO, 25% fat (all food provided)	40% CHO, 45% fat (all food provided)	Acute; ~7 wk
Hu et al., 2014 [19]	China	Parallel (inpatient)	140; 140	Quality	Low-GI staple diet	White-rice diet	~4 days
Ma et al., 2015 [20]	China	Parallel	95; 83	Quality/GL	Intensive low-GL diet	Usual care	~12-14 wk
Trout et al., 2016 [22]	USA	Parallel	68; 68 (ITT)	Quantity	35-40% energy from CHO	50-55% energy from CHO	To delivery
Dodesini et al., 2021 [23]	Italy	Parallel (pilot)	32; 32	Quality	Low-GI, high-fiber diet	Medium-GI diet	~14-15 wk
Feng et al., 2019 [24]	China	Crossover (single meals)	29; 25	Mixed (food substitution)	42 g pistachios	100 g whole-wheat bread	Single meals
Shahzeidi et al., 2019 [25]	Iran	Parallel	90; 80	Fiber	30 g oat bran + yogurt	Yogurt	4 wk
Mijatovic et al., 2020 [26]	Australia	Parallel	46; 45 (mITT)	Quantity	Target ≤135 g CHO/day	180-200 g CHO/day	6 wk
Barati et al., 2021 [27]	Iran	Parallel	112; 112	Fiber	30 g oat bran/day + standard GDM diet	Standard GDM diet	4 wk
Zhang et al., 2021 [28]	China	Parallel	112; 112	Fiber	9.5 g/day mixed fiber + MNT	MNT	8 wk
Allehdan et al., 2022 [29]	Jordan	Parallel (3-arm)	75; 70	Mixed (education/pattern)	CHO counting, or CHO counting + DASH	Control (all receiving metformin)	10-12 wk
Trout et al., 2022 (iPRO) [30]	USA	Crossover (controlled feeding)	19; 12 paired	Quantity/protein	35% CHO, 30% protein	50% CHO, 15% protein	2 × 36 h
Sanpawithayakul et al., 2023 [32]	Thailand	Parallel	96; 96	Quality	Low-to-medium-GI rice (GI 56.9)	Higher-GI rice (GI 80.1)	To insulin decision/delivery
Markussen et al., 2023/2025 (eMOM) [33,37]	Finland	Crossover, then parallel	42; 36 CGM, 32 delivery	Mixed (pattern/quantity)	Plant-based Healthy Nordic diet	Moderately CHO-restricted diet	3 days per period; ~8 wk
Hernandez et al., 2023 (CHOICE) + companions [31,34,40,41,42]	USA	Parallel (provided diet)	59; 46	Mixed	60% complex CHO, 25% fat (all food provided)	40% CHO, 45% fat (all food provided)	7-8 wk
Duraiswamy et al., 2024 [35]	India	Parallel	224; 219	Quality/staple	200 g millet once daily + enhanced MNT	Enhanced MNT	1 month
Yang et al., 2024 [36]	China	Parallel (two-center)	112; 101	Mixed (formula)	Diabetes-specific formula replacing milk + diet	Diet alone	~10 wk, to delivery

Implementation and Achieved Dietary Separation

Prescribed targets were often not reflected in achieved intake. Moses et al. achieved a GI separation of about eight units with a similar carbohydrate percentage; only 20% of the lower-carbohydrate group in the study by Mijatovic et al. reached the target of ≤135 g/day, with an unintended fall in energy intake; and Trout et al. achieved almost no separation (approximately 178 versus 180 g/day), with site-level social differences influencing outcomes more than diet [12,22,26]. During the eMOM parallel phase, the groups differed by under two percentage points of energy from carbohydrate (42.4 ± 7.3% versus 40.5 ± 6.9%) - so the contrast reflected food quality and protein rather than amount - whereas the fully provided CHOICE diets maintained a 60% versus 40% separation with a corresponding difference in fat [18,34,37].

Rescue treatment and selective post-randomization inclusion further altered the observed contrasts. Comparator participants who met insulin-start criteria were switched to the low-GI arm in the studies by Moses et al. and Sanpawithayakul et al., and medication starters or complicated pregnancies were excluded from the delivery cohorts in the studies by Afaghi et al., Hernandez et al., and Markussen et al. These features reduced comparability and explain why outcomes that appeared similar were not combined automatically [16,32,34,37].

Risk of Bias

A single overall risk-of-bias judgment could not fully represent the methodological limitations across all outcomes within each study [7]. Most outcomes were found to raise some concerns, whereas a high risk of bias was common when participants were excluded after treatment escalation, pregnancy complications, or device failure; when completion rates in crossover trials were low; or when data on achieved dietary exposure were unavailable [16,20,25,30]. Objective assessment using laboratory measures or continuous glucose monitoring reduced concerns related to outcome measurement, but it did not overcome the bias from participant selection or missing data [26]. Older trials sometimes lacked accessible protocols or prospective registration, and this limited the assessment of selective outcome reporting [9,11]. Table 2 summarizes the overall pattern, and Table 3 gives the domain-level RoB 2 judgment for every trial.

**Table 2:** Risk-of-bias pattern across the evidence set Risk of bias was assessed using the revised Cochrane risk-of-bias tool for randomized trials (RoB 2). The table summarizes the predominant judgment patterns and representative trial families; detailed domain-specific judgments for each trial are presented in Table 3. CGM, continuous glucose monitoring

Judgment pattern	Examples	Principal basis
Low risk for selected results	Mijatovic ketone result [26]; some objective CGM or laboratory results in well-retained trials [27,28,35]	Prespecified objective measurement with minimal missingness
Some concerns (most trial families)	Louie et al., 2013 [14]; Cypryk et al. [11]; Moses et al. [12]; Louie et al., 2011 [13]; Moreno-Castilla et al. [17]; Hu [19]; Dodesini et al. [23]; Lauszus et al. [10]; Feng et al. [24]; Barati et al. [27]; Zhang et al. [28]; Allehdan et al. [29]; Duraiswamy et al. [35]; Sanpawithayakul et al. [32]; Yang [36]	Unclear concealment or protocol availability, open intervention, incomplete prespecification, or limited carryover assessment
High risk for affected outcomes	Nolan [9]; Afaghi et al. [16]; Ma et al. [20]; Shahzeidi et al. [25]; Trout et al. (iPRO) [30]; CHOICE pilot/large [18,21,34] and eMOM delivery subsets [37]; selected Mijatovic [26] and Trout 2016 [22] outcomes	Attrition, switching, weak achieved contrast, selective completer/subset analysis, or exclusion of medication starters/complications

**Table 3:** Domain-specific risk of bias of the included trials assessed with the revised Cochrane risk-of-bias tool (RoB 2) Judgments are for each trial's principal maternal glycemic or insulin outcome; crossover trials were additionally assessed for period and carryover effects. Domains: D1, randomization process; D2, deviations from intended interventions; D3, missing outcome data; D4, measurement of the outcome; D5, selection of the reported result Ratings: L, low risk; S, some concerns; H, high risk

Trial family (references)	D1	D2	D3	D4	D5	Overall
Nolan 1984 [9]	S	S	H	L	S	H
Lauszus et al., 2001 [10]	S	S	L	L	S	S
Cypryk et al., 2007 [11]	S	S	L	L	S	S
Moses et al., 2009 [12]	S	S	L	L	S	S
Louie et al., 2011/2015 [13,15]	S	L	S	L	S	S
Louie et al., 2013 [14]	S	L	S	L	S	S
Afaghi et al., 2013 [16]	S	H	H	L	S	H
Moreno-Castilla et al., 2013 [17]	L	S	S	L	S	S
Hernandez et al., 2014/2016 [18,21,38,39]	L	S	H	L	S	H
Hu et al., 2014 [19]	S	S	L	L	S	S
Ma et al., 2015 [20]	H	S	H	L	S	H
Trout et al., 2016 [22]	S	H	L	L	S	H
Dodesini et al 2021 [23]	S	S	S	L	S	S
Feng et al., 2019 [24]	S	L	S	S	S	S
Shahzeidi et al., 2019 [25]	S	S	H	L	S	H
Mijatovic et al., 2020 [26]	S	H	S	L	S	H
Barati et al., 2021 [27]	S	L	L	L	S	S
Zhang et al., 2021 [28]	S	L	L	L	S	S
Allehdan et al., 2022 [29]	S	S	S	L	S	S
Trout et al., 2022 (iPRO) [30]	S	S	H	L	S	H
Sanpawithayakul et al., 2023 [32]	L	S	L	L	S	S
Markussen et al., 2023/2025 [33,37]	L	S	H	L	S	H
Hernandez et al., 2023 [31,34,40,41,42]	L	S	H	L	S	H
Duraiswamy et al., 2024 [35]	L	L	L	S	S	S
Yang et al., 2024 [36]	S	S	S	L	S	S

Maternal Glycemia: Acute and Short-Crossover Evidence

Acute and brief crossover studies showed immediate metabolic responses but no evidence that they were sustained or clinically important. A macronutrient-matched low-GI breakfast shifted the post-breakfast glucose curve and peak timing in eight paired participants [14], and a 42-g pistachio meal produced smaller glucose and insulin excursions and a higher late glucagon-like peptide-1 response than 100 g of whole-wheat bread in 25 women, though the meals also differed in fat and protein [24]. In the iPRO crossover, a 35%-carbohydrate/30%-protein condition reduced the three-hour glucose area under the curve versus control (P = 0.01) among the 12 of 19 women completing both 36-hour admissions, limited by attrition and the protein change [30]. Crossover work by Nolan and the CHOICE and eMOM investigators likewise showed short-term responses without delivery or longer-term data [9,18,33].

Maternal Glycemia: Longer Quality and Fiber Interventions

Low-GI staple replacement improved selected postprandial measures [12,19,32]. Duraiswamy et al. randomized 224 women to 200 g of millet daily or enhanced MNT: the gain in composite glycemic control was not significant (11.6 percentage points; P = 0.083), but mean postprandial glucose fell modestly (4.55 mg/dL; P = 0.025), a meal-specific effect rather than superior overall control [35]. Fiber supplements were more consistent for short-term glucose than for clinical outcomes: oat bran lowered fasting and postprandial glucose (Barati et al.; Shahzeidi et al., completer-only), and a 9.5 g/day mixed-fiber supplement improved weekly two-hour control and 42-day postpartum oral glucose tolerance test (OGTT) classification (Zhang et al.), all as additions to MNT rather than effects of GI alone [25,27,28]. In metformin-treated women, carbohydrate counting with or without a DASH pattern lowered fasting and one-hour post-meal glucose versus control at similar carbohydrate grams (P = 0.021 and P < 0.001), and thus the contrast concerned counting and quality rather than quantity [29].

Maternal Glycemia: Quantity and Mixed Provided-Diet Interventions

Lowering carbohydrate quantity did not consistently improve glycemia: Moreno-Castilla et al. found identical insulin use and no glycemic advantage at 40% versus 55%, Trout et al. found no glucose difference amid negligible achieved separation, and Mijatovic et al.'s lower target did not clearly help while unintentionally cutting energy [17,22,26]. In eMOM, three-day moderate restriction lowered fasting insulin and homeostasis model assessment of insulin resistance (HOMA-IR) more than the Healthy Nordic period, but the longer phase showed no difference and no carbohydrate separation [33,37].

CHOICE was the best-controlled demonstration that a higher-complex-carbohydrate, lower-fat diet can meet GDM targets: overall CGM control, OGTT-derived insulin sensitivity, and newborn adiposity were broadly similar to the 40% diet, with higher nocturnal but favorable daytime glucose in companion reports; because amount, complex-food content, and fat moved together, it does not isolate quality from quantity [34,41,42]. The Yang formula trial similarly lowered two-hour post-breakfast glucose (P = 0.008) and raised postprandial time in range (P = 0.045) without improving fasting glucose, supporting focused breakfast replacement rather than general restriction [36].

Insulin and Medication Escalation

Two low-GI trials used an exploratory treatment-threshold outcome-crossing prespecified insulin-start criteria with low-GI rescue offered to the comparator: Moses et al. (9/31 versus 19/32) and Sanpawithayakul et al. (3/48 versus 11/48) pooled to RR 0.43 (95% CI 0.25-0.75; I² = 0%; 159 participants) (Figure 2, panel A) [12,32]. This reflects threshold crossing, not final treatment, because rescue switching reduced actual insulin initiation to 9 versus 10 and 3 versus 1, reversing the apparent contrast; the estimate should be read only as an exploratory effect on threshold crossing, not on medication exposure [12,32]. For lower-carbohydrate assignment, insulin initiation was 2/15 versus 1/15 [11], 41/75 versus 41/75 [17], and about 3/37 versus 2/31 [22], pooling to RR 1.02 (95% CI 0.76-1.35; I² = 0%; 248 participants) (Figure 2, panel B); excluding Trout et al., which achieved no carbohydrate separation, left this essentially unchanged (RR 1.01, 95% CI 0.76-1.35). With only two or three small trials per pool, I² = 0% does not establish the absence of clinical heterogeneity. Separately, Allehdan et al. reduced insulin need with carbohydrate counting with or without DASH (P = 0.026), and Yang et al. did not change the proportion using insulin (16% versus 18%) but delayed initiation (P = 0.015) and shortened exposure (P = 0.024); these distinct outcomes were not combined [29,36].

**Figure 2:**
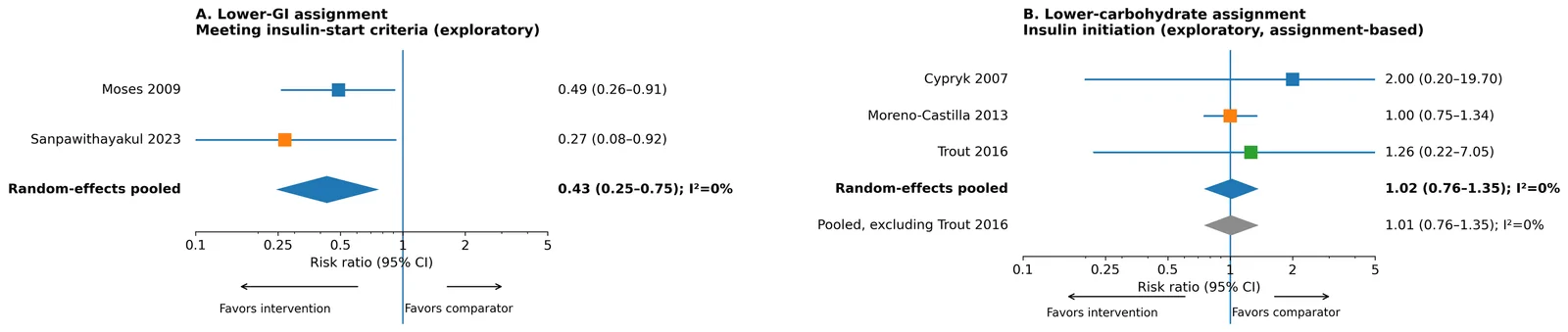
Limited exploratory pooling of insulin-related outcomes Panel A pools Moses 2009 [12] and Sanpawithayakul 2023 [32] (proportion meeting insulin-start criteria, a treatment-threshold outcome, not final insulin use after rescue switching). Panel B pools Cypryk 2007 [11], Moreno-Castilla 2013 [17], and Trout 2016 [22] (assignment-based insulin initiation). In each panel. a filled square marks an individual trial's risk ratio (RR) and the horizontal line its 95% confidence interval (CI); a blue diamond marks the random-effects pooled estimate, with the diamond width representing its 95% CI. In panel B, the lower gray diamond is the sensitivity analysis excluding Trout 2016 (which achieved essentially identical carbohydrate intake between groups). Random-effects DerSimonian-Laird models were used; an RR below 1 favors the intervention. Because each pool contained only two or three small trials, I² = 0% should not be interpreted as an absence of clinical heterogeneity. CI, confidence interval; GI, glycemic index; RR, risk ratio

Principal Findings

The 25 studies were sufficiently informative for a systematic review but were too heterogeneous in intervention construct, achieved exposure, and outcomes for a broad meta-analysis. The evidence does not show that carbohydrate quality is superior to carbohydrate quantity or that greater restriction improves treatment needs or infant outcomes. Lower-GI, fiber, and staple-quality interventions improved selected glycemic and postprandial outcomes, whereas lower-carbohydrate assignment did not reduce insulin initiation. Birth-size and neonatal-adiposity findings were generally neutral but imprecise [11,12,17,25,27,28,32,34,35]. In the pooled analyses, lower-GI assignment reduced crossing of insulin-initiation thresholds, although rescue treatment attenuated the difference in actual insulin use [12,32].

Differences Among Dietary Interventions Affect Interpretation

Dietary trials evaluate substitutions, not isolated nutrients: lowering carbohydrate raises another macronutrient, fiber alters food structure and fermentation, pistachios for bread also change fat, protein, and incretins, a DASH pattern changes minerals and food groups, and CHOICE changed amount, complexity, and fat together; thus, treating them as equivalent yields an average effect of unclear meaning [10,18,24,29]. The gap between prescribed and achieved diets matters as much: near-identical achieved intakes in Trout et al.’s study and eMOM mean that their null assignment-based findings are not physiological equivalence, whereas fully provided CHOICE meals traded generalizability for internal validity [22,34,37].

Treatment Escalation, Attrition, and Outcome Selection

Medication rescue, though necessary clinically, changes interpretation: switching the comparator to a low-GI diet after a treatment threshold turns the trial into a stepped-care comparison rather than a test of the assigned diets [12,32], and excluding medication starters, preterm births, or complicated pregnancies can bias infant-outcome estimates [34,37]. Future trials should retain all randomized participants, define the target treatment effect, and report treatment-policy alongside per-protocol analyses based on achieved adherence [7].

Clinical Implications

Clinical interpretation of these findings should be done cautiously. The available evidence suggests three practical principles. First, carbohydrate intake should remain nutritionally adequate during pregnancy. The commonly recommended minimum intake of 175 g/day applies to pregnancy generally and is not a threshold established by the randomized GDM trials reviewed here [1,2]. Within GDM populations, the available trials did not show a consistent clinical advantage from greater carbohydrate restriction [17,22,26]. Second, lower-GI, intact, fiber-rich carbohydrate sources may help reduce postprandial glucose excursions and progression to treatment thresholds without necessarily lowering total carbohydrate [12,27,28,32,35]. Third, clinicians should monitor the diet actually consumed, including energy intake, fat or protein substitution, ketones when indicated, glucose levels, gestational weight gain, and fetal growth, rather than relying only on the prescribed target [22,26,37]. Formula, staple-substitution, and fiber approaches may help selected patients but should not be considered interchangeable [35,36].

Strengths and Limitations

Strengths included database-specific searches, reconciliation of citations against full-text reports, separation of 34 reports into 25 trial families, exclusion of prior meta-analyses as primary evidence, outcome-level RoB 2, and retention of original-report errors; both prescribed and achieved intake were extracted, two human reviewers made all substantive judgments with artificial intelligence assisting only extraction, and the appendices provide the full search strategies, record flow, and GRADE profile [3,6,7].

Several limitations should be considered when interpreting the findings. Five potentially eligible reports could not be retrieved in full text despite standard retrieval attempts, including database and publisher links and institutional library access. These reports were older or small, and none was expected to provide an eligible carbohydrate comparison suitable for pooling. Their absence is unlikely to change the overall conclusions, although this cannot be ruled out. Google Scholar was used only as a supplementary source because its results could not be exported reproducibly. Five of the 34 included reports were conference abstracts; all five were companion reports of the CHOICE program, and none contributed to either pooled estimate. Full peer-reviewed reports were distinguished from abstracts in Table 1 and in the reference list, and thus the quantitative findings would be unchanged by their exclusion [38-42].

Diagnostic criteria and treatment thresholds differed across countries and across the decades in which the studies were conducted. Other maternal and clinical factors, including pre-existing metabolic comorbidities, maternal adiposity, baseline insulin resistance, concomitant medications, and pregnancy-related physiological and hormonal changes, may also influence glycemic responses to dietary interventions. These factors were inconsistently reported across the included trials and could not be evaluated or adjusted for systematically. Dietary intake was usually reported by participants, and blinding was rarely feasible. Study protocols were often unavailable, and the trials generally lacked sufficient statistical power to assess uncommon maternal or fetal adverse outcomes. Authors were not contacted to obtain missing paired-variance estimates or individual participant data. Finally, the review was not registered prospectively; its absence means that decisions about eligibility grouping, outcome selection, and the limited pooling could not be verified against a pre-existing protocol and may raise concern about selective outcome or reporting decisions. To mitigate this, the full search strategies and record flow (Appendix A), the GRADE evidence profile (Appendix B), and domain-level risk of bias (Table 3) are reported in full, and the extended extraction record is available on request so that readers can independently assess reproducibility.

Future Directions

Future trials should separate the independent effects of carbohydrate quantity, GI, fiber, and the accompanying fat and protein changes, ideally with factorial or controlled-feeding designs, and should prespecify intended and achieved carbohydrate (grams and percentage of energy), GI, GL, fiber, energy, and practical adherence measures [18,22,30,35,37]. A consistent outcome set should include fasting and standardized post-meal glucose, continuous glucose monitoring time in range for pregnancy, medication initiation and dose, beta-hydroxybutyrate, gestational weight gain, hypertensive disorders, preterm birth, large- and small-for-gestational-age, macrosomia, neonatal hypoglycemia, and infant adiposity at a common timepoint [1].

## Conclusions

Randomized evidence on dietary carbohydrate modification in GDM supports a rigorous systematic review but is too varied in design, achieved exposure, and outcome assessment for a broad meta-analysis. Lower-GI strategies reduced crossing of insulin-initiation thresholds in two rescue-permitting trials, whereas lower-carbohydrate assignment did not reduce insulin initiation; selected quality, fiber, and food-substitution interventions improved selected glycemic and postprandial outcomes, but benefits for obstetric outcomes, birth size, and neonatal adiposity were not established. The apparent benefit of carbohydrate quality therefore relates primarily to glycemic and postprandial measures rather than established maternal or neonatal clinical outcomes. The evidence does not support replacing attention to quantity with quality, but rather prioritizing carbohydrate quality within an individualized, nutritionally adequate prescription, with monitoring of actual intake and clinical response.

## Appendices

Appendix A: search strategies and record flow

Databases were searched from inception to July 19, 2026, with no language or date limits. The exact strategies, displayed yields, and derivation of the PRISMA counts are reproduced below.

PubMed/MEDLINE (searched on July 19, 2026; yield: 616 records)

( "Diabetes, Gestational"[Mesh] OR (gestational[tiab] AND diabet*[tiab]) OR GDM[tiab] ) AND ( "Diet, Carbohydrate-Restricted"[Mesh] OR "Dietary Carbohydrates"[Mesh] OR "Glycemic Index"[Mesh] OR "Dietary Fiber"[Mesh] OR ( (carbohydrat[tiab] OR "glycemic index"[tiab] OR "glycaemic index"[tiab] OR "glycemic load"[tiab] OR "glycaemic load"[tiab] OR "low GI"[tiab] OR "high GI"[tiab] OR fiber[tiab] OR fibre[tiab] OR wholegrain[tiab] OR "whole grain"[tiab] OR starch[tiab]) AND (diet[tiab] OR meal[tiab] OR food[tiab] OR nutrition[tiab] OR breakfast[tiab] OR lunch[tiab] OR dinner[tiab]) ) ) NOT (animals[mh] NOT humans[mh])

**Table 4:** Cochrane Central Register of Controlled Trials (CENTRAL; searched on July 19, 2026)

Line	Statement	Displayed yield
#1	MeSH descriptor Diabetes, Gestational, explode all trees	71
#2	(gestational NEXT diabet*):ti,ab,kw	4,403
#3	GDM:ti,ab,kw	2,332
#4	#1 OR #2 OR #3	4,603
#5	MeSH descriptor Diet, Carbohydrate-Restricted, explode all trees	12
#6	MeSH descriptor Dietary Carbohydrates, explode all trees	65
#7	MeSH descriptor Glycemic Index, explode all trees	100
#8	MeSH descriptor Dietary Fiber, explode all trees	74
#9	(carbohydrat* OR "glycemic index" OR "glycaemic index" OR "glycemic load" OR "glycaemic load" OR "low GI" OR "high GI" OR fiber* OR fibre* OR wholegrain* OR "whole grain" OR starch*):ti,ab,kw	45,396
#10	(diet* OR meal* OR food* OR nutrition* OR breakfast* OR lunch* OR dinner*):ti,ab,kw	2,19,083
#11	#5 OR #6 OR #7 OR #8 OR (#9 AND #10)	25,458
#12	#4 AND #11	356

Supplementary Discovery (Other Methods; Nine Unique Records)

Backward and forward citation searching, known-item searches, and reference checking of eligible reports and of Wong et al. [3] identified nine unique additional records. Google Scholar was used only for supplementary discovery because its headline estimates were unstable, and complete bulk export was unavailable; it contributed no additional included studies beyond those found by the methods above.

**Table 5:** PRISMA 2020 record flow (derivation of counts)

PRISMA item	n	Derivation
Database records	972	PubMed 616 + CENTRAL trials 356
Records from other methods	9	Unique citation/known-item records
Records before deduplication	981	972 + 9
Duplicates removed	125	Bibliographic report duplicates
Records screened	856	981 − 125
Title/abstract exclusions	807	856 − 49
Reports sought for retrieval	49	Retrieval queue
Reports not retrieved	5	Full text unavailable
Reports assessed for eligibility	44	49 − 5
Reports excluded	10	Wrong population 3; wrong intervention 3; wrong design 1; ineligible comparison 1; ongoing/no results 2
Reports included	34	44 − 10
Independent randomized studies	25	34 reports mapped to trial families

Appendix B: GRADE evidence profile

Certainty of evidence was assessed with the GRADE approach for the two exploratory pooled insulin-related outcomes. Randomized trials began at high certainty and were downgraded for the domains shown.

**Table 6:** GRADE evidence profile Both pooled analyses were exploratory, and randomized trials began at high certainty. Because each pool contained fewer than 10 studies, I² = 0% was not interpreted as evidence of an absence of clinical heterogeneity, and statistical tests for publication bias were not performed. CI, confidence interval; GI, glycemic index; GRADE, Grading of Recommendations Assessment, Development and Evaluation; RCT, randomized controlled trial

Outcome (studies; participants)	Risk of bias	Inconsistency	Indirectness	Imprecision	Publication bias	Certainty	Plain-language summary
Meeting insulin-start criteria: lower GI (2 RCTs; 159)	Serious (−1): rescue switching, attrition, open interventions	Not downgraded; inconsistency not assessable with only 2 trials (I² uninformative)	Not serious (defined as the treatment-threshold outcome of crossing insulin-start criteria, not a surrogate for actual insulin use)	Serious (−1): few events	Undetected (not assessable, <10 trials)	Low	Lower-GI assignment reduced crossing of insulin-start criteria, but rescue attenuated actual insulin use
Insulin initiation: lower carbohydrate (3 RCTs; 248)	Serious (−1): open interventions, subset analyses	Not downgraded; inconsistency not assessable with only 3 trials (I² uninformative)	Serious (−1): assignment-based; weak achieved carbohydrate separation	Serious (−1): few events; wide CI	Undetected (not assessable, <10 trials)	Very low	No evidence that lower-carbohydrate assignment reduced insulin initiation; robust to excluding the failed-contrast trial (Trout 2016)

Appendix C

**Table 7:** Detailed main findings of the included randomized trial families Construct denotes the primary carbohydrate exposure the trial was designed to test: quality, quantity, or a mixed/whole-diet change. Main findings are summarized from the study-level information used in the manuscript Table 1. AUC, area under the curve; CHO, carbohydrate; CGM, continuous glucose monitoring; DASH, Dietary Approaches to Stop Hypertension; GI, glycemic index; GL, glycemic load; GLP-1, glucagon-like peptide-1; HOMA-IR, homeostasis model assessment of insulin resistance; MUFA, monounsaturated fatty acid; OGTT, oral glucose tolerance test; PROM, premature rupture of membranes

Trial family (references)	Construct	Main findings
Nolan 1984 [9]	Mixed	Improved glucose tolerance on the high-carbohydrate/high-fiber diet; more bowel symptoms; very small mechanistic study
Lauszus et al., 2001 [10]	Quantity/substitution	No metabolic advantage of high-MUFA; greater 24-hour diastolic blood pressure on the high-carbohydrate diet
Cypryk et al., 2007 [11]	Quantity	Similar glycemia and insulin use; little hunger and no ketonuria on the lower-carbohydrate diet
Moses et al., 2009 [12]	Quality	Fewer met insulin-start criteria on low-GI; comparator rescued with low-GI, equalizing actual insulin use
Louie et al., 2011/2015 [13,15]	Quality	No clear differences in pregnancy or 3-month postpartum outcomes; high follow-up attrition (high risk of bias)
Louie et al., 2013 [14]	Quality	Lower and later post-breakfast glucose peak with the low-GI meal
Afaghi et al., 2013 [16]	Quality/fiber	Differential withdrawal and self-selected switching between arms; high risk of bias
Moreno-Castilla et al. 2013 [17]	Quantity	Identical insulin use and no glycemic advantage; less post-randomization weight gain (attenuated after adjustment)
Hernandez et al. 2014/2016 (CHOICE pilot) [18,21,38,39]	Mixed	Higher-complex-CHO diet met glucose targets and lowered postprandial lipids, adipose lipolysis, and inflammatory-gene expression
Hu et al., 2014 [19]	Quality	Lower postprandial glucose with low-GI staples; short exposure
Ma et al., 2015 [20]	Quality/GL	Improved glycemia and lipids; no allocation concealment and exclusions (high risk of bias)
Trout et al., 2016 [22]	Quantity	No glucose or infant-outcome difference; achieved CHO approximately 178 vs. 180 g/day (no separation); strong site effects
Dodesini et al., 2021 [23]	Quality	Improved glycemia; small study; birthweight denominator discrepancy
Feng et al., 2019 [24]	Mixed (food substitution)	Smaller glucose/insulin excursions and higher late GLP-1 with pistachios; meals not macronutrient-matched
Shahzeidi et al., 2019 [25]	Fiber	Lower fasting glucose and better gestational weight control with oat bran; completer-only analysis
Mijatovic et al., 2020 [26]	Quantity	Only 20% reached target; no clear glycemic benefit; unintended energy restriction; ketones low risk of bias
Barati et al., 2021 [27]	Fiber	Lower fasting and postprandial glucose with oat bran
Zhang et al., 2021 [28]	Fiber	Better weekly 2-hour glucose and 42-day postpartum OGTT; fewer cases of PROM and neonatal hyperbilirubinemia
Allehdan et al., 2022 [29]	Mixed (education/pattern)	Lower fasting and 1-hour glucose and less insulin need with counting +/- DASH at similar CHO grams
Trout et al., 2022 (iPRO) [30]	Quantity/protein	Lower 3-hour glucose AUC with lower-CHO/higher-protein; 37% did not complete both periods
Sanpawithayakul et al. 2023 [32]	Quality	Fewer met insulin-start criteria on low-GI rice; comparator rescued, equalizing actual insulin use
Markussen et al., 2023/2025 (eMOM) [33,37]	Mixed (pattern/quantity)	Short-term restriction lowered fasting insulin/HOMA-IR; parallel phase achieved CHO 42.4% vs. 40.5% (little separation); medication starters excluded from delivery analysis
Hernandez et al. 2023 (CHOICE) + companions [31,34,40,41,42]	Mixed	Similar overall CGM control, insulin sensitivity, and newborn adiposity; higher nocturnal but favorable daytime glucose; altered infant microbiome
Duraiswamy et al., 2024 [35]	Quality/staple	Nonsignificant gain in composite control (P = 0.083); modest fall in mean postprandial glucose (P = 0.025); good retention
Yang et al., 2024 [36]	Mixed (formula)	Lower 2-hour post-breakfast glucose and higher postprandial time in range; delayed and shortened insulin use; fasting glucose unchanged

## References

[1] (2026). 15. Management of Diabetes in Pregnancy: Standards of Care in Diabetes-2026. Diabetes Care.

[2] Institute of Medicine (2005). Dietary Reference Intakes for Energy, Carbohydrate, Fiber, Fat, Fatty Acids, Cholesterol, Protein, and Amino Acids.

[3] Wong MM, Yuen-Man Chan M, Ng TP, Louie JC (2024). Impact of carbohydrate quantity and quality on maternal and pregnancy outcomes in gestational diabetes mellitus: A systematic review and meta-analysis. Diabetes Metab Syndr.

[4] Page MJ, McKenzie JE, Bossuyt PM (2021). The PRISMA 2020 statement: an updated guideline for reporting systematic reviews. BMJ.

[5] Higgins JPT, Thomas J, Chandler J, Cumpston M, Li T, Page MJ (2024). Cochrane Handbook for Systematic Reviews of Interventions version 6.5. https://www.cochrane.org/handbook.

[6] Campbell M, McKenzie JE, Sowden A (2020). Synthesis without meta-analysis (SWiM) in systematic reviews: reporting guideline. BMJ.

[7] Sterne JA, Savović J, Page MJ (2019). RoB 2: a revised tool for assessing risk of bias in randomised trials. BMJ.

[8] Guyatt GH, Oxman AD, Vist G (2011). GRADE guidelines: 4. Rating the quality of evidence--study limitations (risk of bias). J Clin Epidemiol.

[9] Nolan CJ (1984). Improved glucose tolerance in gestational diabetic women on a low fat, high unrefined carbohydrate diet. Aust N Z J Obstet Gynaecol.

[10] Lauszus FF, Rasmussen OW, Henriksen JE, Klebe JG, Jensen L, Lauszus KS, Hermansen K (2001). Effect of a high monounsaturated fatty acid diet on blood pressure and glucose metabolism in women with gestational diabetes mellitus. Eur J Clin Nutr.

[11] Cypryk K, Kamińska P, Kosiński M, Pertyńska-Marczewska M, Lewiński A (2007). A comparison of the effectiveness, tolerability and safety of high and low carbohydrate diets in women with gestational diabetes. Endokrynol Pol.

[12] Moses RG, Barker M, Winter M, Petocz P, Brand-Miller JC (2009). Can a low-glycemic index diet reduce the need for insulin in gestational diabetes mellitus? A randomized trial. Diabetes Care.

[13] Louie JC, Markovic TP, Perera N, Foote D, Petocz P, Ross GP, Brand-Miller JC (2011). A randomized controlled trial investigating the effects of a low-glycemic index diet on pregnancy outcomes in gestational diabetes mellitus. Diabetes Care.

[14] Louie JC, Markovic TP, Ross GP, Foote D, Brand-Miller JC (2012). Timing of peak blood glucose after breakfast meals of different glycemic index in women with gestational diabetes. Nutrients.

[15] Louie JC, Markovic TP, Ross GP, Foote D, Brand-Miller JC (2015). Effect of a low glycaemic index diet in gestational diabetes mellitus on post-natal outcomes after 3 months of birth: a pilot follow-up study. Matern Child Nutr.

[16] Afaghi A, Ghanei L, Ziaee A (2013). Effect of low glycemic load diet with and without wheat bran on glucose control in gestational diabetes mellitus: A randomized trial. Indian J Endocrinol Metab.

[17] Moreno-Castilla C, Hernandez M, Bergua M (2013). Low-carbohydrate diet for the treatment of gestational diabetes mellitus: a randomized controlled trial. Diabetes Care.

[18] Hernandez TL, Van Pelt RE, Anderson MA (2014). A higher-complex carbohydrate diet in gestational diabetes mellitus achieves glucose targets and lowers postprandial lipids: a randomized crossover study. Diabetes Care.

[19] Hu ZG, Tan RS, Jin D, Li W, Zhou XY (2014). A low glycemic index staple diet reduces postprandial glucose values in Asian women with gestational diabetes mellitus. J Investig Med.

[20] Ma WJ, Huang ZH, Huang BX (2015). Intensive low-glycaemic-load dietary intervention for the management of glycaemia and serum lipids among women with gestational diabetes: a randomized control trial. Public Health Nutr.

[21] Hernandez TL, Van Pelt RE, Anderson MA (2016). Women with gestational diabetes mellitus randomized to a higher-complex carbohydrate/low-fat diet manifest lower adipose tissue insulin resistance, inflammation, glucose, and free fatty acids: a pilot study. Diabetes Care.

[22] Trout KK, Homko CJ, Wetzel-Effinger L (2016). Macronutrient composition or social determinants? impact on infant outcomes with gestational diabetes mellitus. Diabetes Spectr.

[23] Dodesini AR, Donadoni V, Ciriello E (2021). Effects of a low glycemic index high in fiber diet on blood glucose in women with gestational diabetes. J Matern Fetal Neonatal Med.

[24] Feng X, Liu H, Li Z, Carughi A, Ge S (2019). Acute effect of pistachio intake on postprandial glycemic and gut hormone responses in women with gestational diabetes or gestational impaired glucose tolerance: a randomized, controlled, crossover study. Front Nutr.

[25] Shahzeidi M, Nadjarzadeh A, Rahmanian M, Salehi Abargouei A, Fallahzadeh H, Mogibian M (2019). The effect of oat bran supplement on fasting blood sugar and glycosylated hemoglobin in patients with gestational diabetes mellitus. J Nutr Food Secur.

[26] Mijatovic J, Louie JC, Buso ME, Atkinson FS, Ross GP, Markovic TP, Brand-Miller JC (2020). Effects of a modestly lower carbohydrate diet in gestational diabetes: a randomized controlled trial. Am J Clin Nutr.

[27] Barati Z, Iravani M, Karandish M, Haghighizadeh MH, Masihi S (2021). The effect of oat bran consumption on gestational diabetes: a randomized controlled clinical trial. BMC Endocr Disord.

[28] Zhang Z, Li J, Hu T, Xu C, Xie N, Chen D (2021). Interventional effect of dietary fiber on blood glucose and pregnancy outcomes in patients with gestational diabetes mellitus. Zhejiang Da Xue Xue Bao Yi Xue Ban.

[29] Allehdan S, Basha A, Hyassat D, Nabhan M, Qasrawi H, Tayyem R (2022). Effectiveness of carbohydrate counting and dietary approach to stop hypertension dietary intervention on managing gestational diabetes mellitus among pregnant women who used metformin: a randomized controlled clinical trial. Clin Nutr.

[30] Trout KK, Compher CW, Dolin C, Burns C, Quinn R, Durnwald C (2022). Increased protein with decreased carbohydrate intake reduces postprandial blood glucose levels in women with gestational diabetes: the iPRO study. Womens Health Rep (New Rochelle).

[31] Sugino KY, Hernandez TL, Barbour LA, Kofonow JM, Frank DN, Friedman JE (2022). A maternal higher-complex carbohydrate diet increases bifidobacteria and alters early life acquisition of the infant microbiome in women with gestational diabetes mellitus. Front Endocrinol (Lausanne).

[32] Sanpawithayakul K, Kaewprasert N, Tantiyavarong P (2023). Effects of the consumption of low to medium glycemic index-based rice on the rate of insulin initiation in patients with gestational diabetes: a triple-blind, randomized, controlled trial. Clin Ther.

[33] Markussen LT, Kivelä J, Lindström J, Ashrafi RA, Heinonen S, Koivusalo S, Meinilä J (2023). Short-term effect of plant-based Nordic diet versus carbohydrate-restricted diet on glucose levels in gestational diabetes - the eMOM pilot study. BMC Nutr.

[34] Hernandez TL, Farabi SS, Fosdick BK (2023). Randomization to a provided higher-complex-carbohydrate versus conventional diet in gestational diabetes mellitus results in similar newborn adiposity. Diabetes Care.

[35] Duraiswamy M, Jayaseelan V, Ramakrishnan J, Rengaraj S, Krishnamoorthy Y, Kais M, Subbaiah M (2024). Effect of millets once a day on glycaemic control among women with gestational diabetes mellitus in a tertiary care setting - a randomized controlled trial. Indian J Endocrinol Metab.

[36] Yang R, Han W, Zheng W (2024). Administration of a diabetes-specific formula can improve postprandial glycemic control and delay insulin use in gestational diabetes mellitus: a randomized controlled trial from two centers. Clin Nutr.

[37] Markussen LT, Kivelä J, Lindström J (2025). Glycemic control in women with GDM: insights from a randomized controlled pilot trial on plant-based Nordic healthy diet versus moderately carbohydrate restricted diet. BMC Nutr.

[38] Hernandez TL, Barbour LA, Friedman JJ, Reece MS, Krause MA, Van Pelt RE (2012). Higher carbohydrate versus higher fat diet in gestational diabetes: a pilot study. J Obstet Gynecol Neonatal Nurs.

[39] Hernandez TL (2015). A higher complex carbohydrate diet in gestational diabetes improves maternal metabolic outcomes and infant adiposity: a randomized study. Nurs Outlook.

[40] Hernandez TL, Farabi SS, Arbet J (2020). Randomization to a higher-complex carbohydrate vs conventional diet in GDM improves glucose tolerance and results in similar cord blood insulin and newborn adiposity. Diabetes.

[41] Hernandez TL, Dunn EZ, Hirsch N (2022). Randomization to a higher-complex carbohydrate or conventional diet in GDM results in equivalent glycemic control by continuous glucose monitoring. Diabetes.

[42] Hagen-Lillevik S, Barbour L, Dunn E (2025). A higher complex carbohydrate diet in gestational diabetes lowers daytime glucose and associates with reduced neonatal adiposity. Curr Dev Nutr.

